# Early Treatment of Post-Stroke Spasticity with Botulinum Toxin Type A: Myth or Reality?

**DOI:** 10.3390/toxins18080356

**Published:** 2026-08-21

**Authors:** Alessandro Picelli, Rita Di Censo, Nicola Smania, Valentina Varalta, Mirko Filippetti

**Affiliations:** 1 Department of Neurosciences, Biomedicine and Movement Sciences, University of Verona, 37134 Verona, Italy; nicola.smania@univr.it (N.S.); valentina.varalta@univr.it (V.V.); mirko.filippetti@univr.it (M.F.); 2 Department of Neurosciences, University Hospital of Verona, 37126 Verona, Italy; rita.dicenso@aovr.veneto.it; 3 Canadian Advances in Neuro-Orthopedics for Spasticity Consortium (CANOSC), Kingston, ON K7K 1Z6, Canada

**Keywords:** botulinum toxins, muscle spasticity, therapeutics

## Abstract

Post-stroke spasticity may interfere with function and rehabilitation and contribute to pain, contracture, care burden, and disability. Botulinum toxin type A is an established focal treatment for post-stroke spasticity, but it is often introduced in the chronic phase, when persistent involuntary muscle overactivity may coexist with secondary musculoskeletal complications. This opinion paper critically examines whether early treatment with botulinum toxin should be considered a myth or an emerging clinical reality. Current evidence from meta-analyses, randomized trials, observational cohorts, and pooled analyses was reviewed with particular attention to treatment timing, treatment triggers, functional outcomes, and prevention of secondary complications. The literature supports the clinical usefulness of earlier treatment in appropriately selected patients, particularly for reducing focal involuntary muscle overactivity and resistance to passive movement, delaying symptomatic progression, slowing contracture development, and reducing pain-related complications. However, a consistent additional effect on active motor recovery has not been demonstrated, and evidence for participation and long-term functional independence remains less definitive. Moreover, “early” is heterogeneously defined across studies, ranging from the first weeks to the first year after stroke. We therefore propose moving from a rigid time-based concept of “early” treatment toward timely, target- and goal-guided intervention, integrating botulinum toxin with rehabilitation when a clinically relevant and modifiable focal neural treatment target emerges. To operationalize this concept, we propose a pragmatic set of clinical variables including the neural treatment target, its clinical impact and trajectory, passive musculoskeletal status, pain and care burden, motor recovery context, rehabilitation goals, and patient priorities.

## 1. Introduction

Post-stroke spasticity (PSS) is a common and clinically relevant manifestation of the upper motor neuron syndrome after stroke [1,2,3,4]. Recent consensus work has highlighted the importance of distinguishing spasticity itself from the broader constellation of motor and musculoskeletal abnormalities that may coexist after an upper motor neuron lesion [5]. For the purposes of this article, PSS is considered a disorder of sensorimotor control resulting from upper motor neuron pathology, clinically characterized by velocity- and length-dependent involuntary muscle overactivity that may be intermittent or sustained during passive stretch [5]. This operational definition distinguishes spasticity from other positive and negative manifestations of the upper motor neuron syndrome that may accompany it but are not required to define it.

In clinical practice, however, patients rarely present with spasticity in isolation. Other positive motor phenomena may include spastic dystonia, spasms, involuntary co-contraction, synkinesis, and abnormal synergistic activation, whereas negative motor signs include weakness and impaired selective motor control [5,6,7]. Weakness should therefore not be considered a component of spasticity itself, but rather a negative manifestation of the upper motor neuron syndrome that commonly coexists with involuntary muscle overactivity. Similarly, abnormal co-contraction during voluntary movement refers to simultaneous activation of agonist and antagonist muscles and is conceptually distinct from the stretch-evoked component of spasticity. Over time, these neural impairments may coexist with non-neural peripheral changes, including connective-tissue remodeling, muscle shortening and fibrosis, increased passive stiffness, and muscle or joint contracture [7,8]. The broader umbrella construct termed the “spasticity syndrome” has recently been proposed to encompass neurological signs and symptoms associated with upper motor neuron pathology together with patient-reported symptoms and non-neural peripheral soft-tissue changes [7]. Importantly, use of this umbrella term does not imply that weakness, abnormal co-contraction, or secondary soft-tissue changes are themselves spasticity; identifying the individual components is clinically relevant because they differ in mechanism, functional consequences, and treatment targets.

The reported prevalence of PSS varies considerably across studies, depending on population characteristics, stroke severity, timing of assessment, the definition adopted, and the measurement tools used. Estimates range from 4% to 27% within the first 6 weeks, approximately 19% at 3 months, 21.7% to 42.6% between 4 and 6 months, and 17% to 38% at 12 months after stroke onset [1,9,10]. These estimates should be interpreted cautiously because many studies, particularly those examining early PSS, have operationalized spasticity using the Modified Ashworth Scale (MAS) or similar clinical scales based on resistance to passive movement. The MAS quantifies resistance during passive movement but does not directly measure the neural component of spasticity or reliably distinguish it from non-neural mechanical resistance arising from muscle and soft tissues [7]. Consequently, prevalence estimates based predominantly on such scales should be regarded as operational estimates of clinically detected resistance or hyper-resistance rather than direct measurements of spasticity itself. In patients with paresis, the prevalence of PSS appears substantially higher, and greater initial motor impairment is consistently associated with an increased risk and earlier development of spasticity [5,10].

Early manifestations of spasticity may be transient in some patients; however, clinically relevant spasticity can contribute to complications from the early stages after stroke and does not need to progressively increase over time before becoming problematic. In other patients, persistent or progressive involuntary muscle overactivity may interact with paresis, impaired motor control, immobility, and evolving peripheral tissue changes, contributing to abnormal limb posture, pain, loss of passive range of motion, hygiene and care difficulties, reduced activity and participation, and eventually fixed contracture [1,7,10,11]. Limb deformity should therefore not be attributed to spasticity alone: it may result from secondary musculoskeletal changes in the absence of substantial neural muscle overactivity, or from the combined effects of musculoskeletal changes and spasticity. Accurate identification of the predominant impairments and their evolution over time is therefore essential for appropriate treatment selection and goal setting.

Botulinum toxin type A (BoNT-A) is widely recommended as the first-line focal pharmacological treatment for clinically relevant focal muscle overactivity associated with PSS, with established efficacy in reducing involuntary muscle activation and improving passive function [12,13,14]. Its principal treatment target is therefore the neural component of excessive muscle activation; BoNT-A does not directly restore corticospinal weakness or reverse established fixed structural contracture. This distinction is particularly relevant to treatment timing. In routine clinical practice, BoNT-A is still frequently introduced in the chronic phase of stroke, when persistent neural muscle overactivity may already coexist with secondary biomechanical complications [15,16]. Earlier identification and treatment of a clinically relevant focal neural treatment target could therefore address a modifiable impairment before fixed secondary changes become established and interfere further with rehabilitation. This raises the central question addressed in this opinion paper: should BoNT-A be used earlier in the course of PSS, and, more importantly, should treatment be defined primarily by time from stroke or by the timely emergence of a clinically relevant and modifiable treatment target?

## 2. Rationale for Early Botulinum Toxin Treatment in Post-Stroke Spasticity

The rationale for early BoNT-A treatment in PSS is based primarily on the timely identification and reduction in focal involuntary muscle overactivity. By reducing excessive neural muscle activation, BoNT-A may lessen sustained abnormal posturing when this is driven, at least in part, by focal muscle overactivity. As the post-stroke course evolves, resistance to passive movement may reflect different and changing contributions from neural involuntary muscle activity and non-neural tissue properties, including muscle shortening, fibrosis, altered connective tissue, and contracture [5,6,7,8]. These components should be distinguished because they represent different treatment targets: BoNT-A acts on excessive neural muscle activation but does not directly reverse corticospinal weakness or established structural contracture.

From this perspective, the clinical benefits of BoNT-A extend beyond the immediate reduction in focal involuntary muscle overactivity. In appropriately selected patients, BoNT-A can improve passive function, reduce spasticity-associated pain, and facilitate positioning, hygiene, and care [12,13,14]. When administered early after stroke, randomized evidence also indicates that BoNT-A can slow the loss of passive range of motion and contracture development [17,18]. These effects should not be interpreted as evidence that BoNT-A directly modifies soft tissue. Rather, reducing a neural contributor to abnormal positioning before fixed biomechanical limitations become established may create more favorable conditions for goal-directed rehabilitation and motor practice. This rationale may be particularly relevant during the early post-stroke period, when spontaneous biological recovery is most dynamic; however, experience- and practice-dependent neural plasticity continues throughout the recovery continuum and is not restricted to an early temporal window [19,20].

This potential contribution should nevertheless be interpreted carefully. BoNT-A does not restore corticospinal integrity, directly treat weakness, reverse established non-neural musculoskeletal changes, or constitute an isolated disease-modifying intervention. Its role is to selectively reduce focal involuntary muscle overactivity when this represents a clinically relevant and modifiable treatment target, for example because it interferes with passive or active function, care, positioning, pain, or rehabilitation goals. BoNT-A should therefore be considered one component of an individualized, multidisciplinary, goal-oriented rehabilitation strategy rather than a treatment for the entire upper motor neuron syndrome or for all components of the broader spasticity syndrome [7].

Sensitive identification of the neural component of abnormal resistance is particularly relevant when considering timely treatment. In EUBoSS, patients with severe upper-limb impairment were screened repeatedly using surface electromyography during passive stretch and were randomized when abnormal stretch-related muscle activation was detected within the first 6 weeks after stroke. The Tardieu Scale identified substantially fewer patients than the neurophysiological assessment, indicating that resistance-based clinical scales may fail to detect early abnormal neural muscle activity identifiable electrophysiologically [17]. This finding is clinically relevant because an early increase in resistance to passive movement should not automatically be equated with spasticity; conversely, neurophysiological evidence of abnormal stretch-related muscle activation may help identify a potentially modifiable neural treatment target.

## 3. Current Evidence on Early Botulinum Toxin Treatment

The first meta-analysis specifically addressing early BoNT-A treatment included six randomized studies in which injections were administered within 3 months after stroke. Compared with the control conditions used in the included studies, early BoNT-A significantly reduced clinically measured resistance to passive movement at assessments from 4 to 12 weeks after injection. However, the pooled analyses did not demonstrate statistically significant superiority over control for disability at 4 weeks or active function at 4 and 20–24 weeks, while spasticity-related pain showed only a non-significant trend toward improvement at 4 weeks [15]. Importantly, this meta-analysis compared early BoNT-A with the control conditions of the included trials; it did not compare early with delayed BoNT-A treatment. Thus, these findings do not establish whether early treatment is superior to delayed BoNT-A for disability or active function. Rather, they illustrate a pattern that continues to characterize the literature: effects on clinically measured resistance to passive movement are more consistent than effects on active motor function and broader disability outcomes.

Subsequent randomized studies have strengthened, although not definitively settled, the case for earlier intervention. In the ONTIME randomized pilot trial, a single injection of abobotulinumtoxinA 500 U was administered 2–12 weeks after a first stroke to patients with a MAS score ≥ 2 in a primary targeted upper-limb muscle group; the median time from stroke to treatment was 5.86 weeks [21]. At baseline, all participants fulfilled the MAS criterion, whereas clinically symptomatic spasticity was not required in every patient. Symptomatic spasticity was defined by the presence of at least one clinically relevant feature—pain, involuntary movements, or impaired active or passive function—in addition to increased resistance to passive movement. BoNT-A significantly prolonged the time to fulfillment of re-injection criteria compared with placebo [21]. Thus, ONTIME is particularly relevant to the concept of timely treatment because progression was operationalized using not only a resistance-based clinical measure but also clinically meaningful symptoms or functional consequences.

EUBoSS moved the intervention still earlier and adopted a different treatment trigger. Patients with severe upper-limb impairment were screened repeatedly during the first 6 weeks after a first stroke, and treatment was initiated when abnormal stretch-related muscle activation was detected using surface electromyography during passive movement [17]. This neurophysiological approach is particularly relevant because it sought to identify a neural treatment target rather than relying exclusively on resistance-based clinical scales. BoNT-A reduced EMG-detected abnormal stretch-related muscle activity, slowed contracture development—as reflected by a slower loss of passive range of motion—and reduced the need for splinting [17]. At the fixed onabotulinumtoxinA regimen used in EUBoSS, active arm function did not differ significantly from placebo; importantly, however, there was no evidence that treatment interfered with spontaneous upper-limb functional recovery [17]. This reassuring finding is consistent with the earlier double-blind randomized placebo-controlled phase II pilot study by Cousins et al., in which low-dose BoNT-A was administered within 3 weeks after stroke and no detrimental signal on arm-function recovery was observed [22]. Taken together, these randomized data support an important distinction: early BoNT-A has not demonstrated a consistent additional effect on active motor recovery, but the dosing regimens evaluated in these studies have not shown evidence of interfering with spontaneous upper-limb recovery. A subsequent secondary analysis of EUBoSS further suggested that early treatment may reduce the development of post-stroke upper-limb pain and analgesic use, particularly in patients treated before pain becomes established [18].

Observational and real-world data are broadly consistent with a potential advantage of earlier treatment, although findings are heterogeneous and causal interpretation is limited by study design. The early-BIRD study demonstrated clinical improvement across all treatment-timing groups, with descriptive but non-significant trends favoring earlier BoNT-A administration [23]. A multicenter longitudinal cohort of BoNT-A-naïve patients found a greater reduction in MAS scores when the first injection was performed within 90 days after stroke onset [9]. A subsequent secondary analysis of the same cohort suggested a more favorable motor-recovery trajectory among patients treated within 90 days despite greater baseline impairment. In particular, a significantly faster improvement in Fugl–Meyer Assessment scores was observed at 12 weeks, whereas differences in motor strength and global disability were less definitive [24]. These findings should not be interpreted as evidence that BoNT-A directly treats corticospinal weakness; rather, they suggest that reducing focal involuntary muscle overactivity earlier in the recovery trajectory may be associated with broader rehabilitation-related improvements in selected patients. More recently, a retrospective analysis pooling individual data from five prospective studies found significantly greater improvement in patient-centered goal attainment at 12 weeks in patients treated within the first year after stroke than in those treated at or beyond 1 year [25].

The main differences in study design, eligibility criteria, treatment trigger, post-stroke timing, and timing-related outcomes are summarized in Table 1. These differences are clinically important because “early treatment” has been operationalized using substantially different constructs: chronological windows from stroke, MAS-based entry criteria, neurophysiological detection of abnormal stretch-related muscle activation, symptomatic or functional criteria for progression and re-injection, and retrospective treatment-timing categories. Accordingly, the available literature should not be interpreted as evaluating a single homogeneous concept of “early BoNT-A treatment.” Rather, it encompasses different approaches to identifying when a potentially modifiable neural impairment becomes an appropriate treatment target.

Taken together, the current evidence supports early BoNT-A treatment as an increasingly evidence-based strategy in appropriately selected patients with PSS. The most consistent benefits concern reduction in focal involuntary muscle overactivity and clinically measured resistance to passive movement, delay of symptomatic progression, slowing of passive range-of-motion loss and contracture development, and reduction in pain-related complications. Randomized evidence also provides reassurance that the early BoNT-A dosing regimens studied do not appear to interfere with spontaneous upper-limb motor recovery. However, a consistent additional effect on active motor recovery has not been demonstrated, and evidence for long-term participation, sustained functional independence, and broader patient-centered outcomes remains less definitive. Importantly, the available studies used heterogeneous eligibility criteria, treatment triggers, and definitions of “early” treatment, and observational comparisons according to time from stroke should not be interpreted as randomized early-versus-delayed treatment comparisons. Consequently, the current literature supports the principle of earlier or timely intervention in selected patients more strongly than it supports a single optimal chronological treatment window.

## 4. What Does “Early” Really Mean in the Current Literature?

A major limitation of the current literature is that “early” is not uniformly defined and should not be interpreted as a single established therapeutic window. Across studies, the term has been operationalized primarily according to time elapsed from stroke onset, but the thresholds used vary substantially, ranging from the first days or weeks after stroke to the entire first post-stroke year. In some studies, moreover, chronological timing was combined with clinical or neurophysiological criteria intended to identify the emergence of a potentially modifiable treatment target.

The 2016 meta-analysis by Rosales et al. defined early intervention as BoNT-A administered within 3 months after stroke [15]. As summarized in Table 1, however, individual studies applied markedly different timing criteria and treatment triggers. The pre-EUBoSS pilot trial by Cousins et al. administered low-dose BoNT-A within 3 weeks after stroke [22], whereas ONTIME enrolled and treated patients 2–12 weeks after a first stroke on the basis of a MAS score ≥ 2 in a primary targeted upper-limb muscle group [21]. EUBoSS adopted a different strategy: patients with severe upper-limb impairment were screened repeatedly during the first 6 weeks after stroke and were treated when abnormal stretch-related muscle activation was identified neurophysiologically; the mean time to intervention was 18 days [17]. Thus, in EUBoSS, treatment timing was determined not only by chronology but also by the emergence of a detectable neural treatment target.

Observational studies have used broader and more pragmatic timing definitions. Picelli et al. adopted a 90-day threshold, comparing BoNT-A-naïve patients treated within 90 days after stroke with those treated later but still within 12 months [9,24]. The early-BIRD study categorized patients according to quartiles of time from stroke to first BoNT-A treatment in routine clinical practice rather than applying a single predefined early-treatment threshold [23]. The broadest operational definition was used in the pooled goal-attainment analysis, in which treatment initiated within the first year after stroke was classified as early and treatment at or beyond 1 year as late [25].

Accordingly, “early BoNT-A treatment” in the current literature does not represent a single homogeneous intervention. It may refer to treatment within days or weeks of stroke, within 3 months, within 90 days, or within the first year, and these chronological definitions may coexist with substantially different eligibility criteria and treatment triggers. This heterogeneity limits direct comparison across studies and may contribute to differences in reported outcomes. More importantly, it indicates that the question should not be framed simply as “early versus late,” but rather in relation to the reference point used to define timing: time from stroke onset, time from emergence of the target impairment, or time from development of clinically relevant consequences. This distinction provides the basis for moving from a purely chronological concept of “early” treatment toward a more clinically meaningful concept of timely intervention.

## 5. “Early” According to the Stroke Recovery Timeline

One way to conceptualize early BoNT-A treatment is to anchor it to the temporal framework of stroke recovery. According to the Stroke Recovery and Rehabilitation Roundtable (SRRR), the hyperacute phase extends from stroke onset to 24 h, the acute phase from 1 to 7 days, the early subacute phase from 7 days to 3 months, the late subacute phase from 3 to 6 months, and the chronic phase begins beyond 6 months after stroke [19]. This framework was developed as a common temporal convention for stroke recovery research, informed by the evolving biology of recovery, rather than as a definition of a treatment-specific therapeutic window.

This temporal framework is relevant because the rate and biological mechanisms of recovery change over time after stroke. Spontaneous biological recovery begins early and generally tapers over time; for upper-limb motor recovery, the period of most rapid spontaneous change extends over the first weeks to months [19]. The SRRR framework identifies the early post-stroke period, particularly the interval spanning the first week to the first month, as an important phase for studying restorative interventions because recovery-related biological processes are especially dynamic during this time [19]. This temporal pattern of spontaneous recovery should not be equated with a time limit on neural plasticity. Experience- and practice-dependent neural reorganization can occur throughout the recovery continuum, including in the chronic phase, and remains responsive to rehabilitation, behavioral experience, and environmental demands [19,20]. The early subacute phase should therefore be understood as a period characterized by particularly rapid spontaneous recovery and a distinct biological recovery milieu, rather than as an exclusive window in which neural plasticity or treatment-induced reorganization can occur.

When this framework is applied to PSS, the relevance of earlier BoNT-A treatment lies not in the assumption that neural plasticity is available only during the early post-stroke period, but in identifying a clinically relevant focal neural treatment target while spontaneous recovery and secondary musculoskeletal changes are still evolving. Focal involuntary muscle overactivity may emerge during the acute or early subacute phase and coexist with paresis, impaired motor control, abnormal posturing, and evolving non-neural musculoskeletal changes. BoNT-A does not directly enhance neural plasticity or spontaneous biological recovery; rather, by reducing a modifiable component of focal involuntary muscle overactivity before fixed biomechanical limitations become established, treatment may create more favorable conditions for positioning, passive function, pain management, care, and goal-directed rehabilitation.

Importantly, the biological rationale for earlier intervention does not imply that BoNT-A loses relevance once the early subacute phase has passed. In the late subacute and chronic phases, focal involuntary muscle overactivity may remain an appropriate treatment target when it interferes with function, care, positioning, pain, or rehabilitation goals. Likewise, experience- and practice-dependent neural reorganization and functional adaptation may continue well beyond 6 months after stroke [19,20]. Thus, the stroke recovery timeline provides a useful biological context for interpreting treatment timing, but it should not be used in isolation to determine when BoNT-A is indicated. Chronological timing should instead be considered together with the emergence and clinical relevance of the individual treatment target.

## 6. From “Early” to “Timely” Treatment

A clinically useful alternative to defining early treatment solely according to time from stroke is to anchor treatment timing to the emergence of a potentially modifiable focal neural treatment target and to its relevance for the individual patient. Because PSS may emerge at different times after stroke and may vary considerably in persistence, severity, and clinical consequences, treatment that is “early” according to chronology is not necessarily treatment that is appropriately timed. In this context, the concept of timely treatment may be more informative: BoNT-A should be considered when focal involuntary muscle overactivity becomes clinically relevant, interferes with meaningful goals, or is judged likely to contribute to clinically important secondary consequences in a patient at sufficiently high risk.

The natural history of PSS supports this individualized approach. Spasticity may emerge within the first days or weeks after stroke, but its onset and subsequent course are highly variable [1]. Mild or transient abnormalities may not require focal pharmacological treatment, whereas persistent or progressive involuntary muscle overactivity may contribute to abnormal positioning, pain, loss of passive range of motion, difficulties with care or hygiene, interference with rehabilitation, and secondary musculoskeletal changes [1,10]. Early recognition of patients at increased risk is therefore important [26]. However, the presence of spasticity—or an isolated increase in resistance to passive movement—should not in itself mandate BoNT-A treatment.

Clinical relevance should therefore not be defined by a resistance-based score alone. As discussed above, resistance to passive movement may reflect varying neural and non-neural contributions, and the decision to treat should integrate the nature and focality of involuntary muscle overactivity with its current or anticipated consequences. Relevant considerations include interference with passive or active function, pain, positioning, hygiene and care, mobility, progression of passive musculoskeletal restriction, rehabilitation goals, and the risk of secondary complications. This approach is consistent with ICF-based concepts of disabling spasticity, in which clinical significance is determined by effects on body function, activity, and participation [10,11].

The existing randomized trials illustrate different ways in which treatment triggers can be operationalized. In ONTIME, all participants fulfilled a MAS-based entry criterion, whereas symptomatic spasticity was not required at baseline; subsequent re-injection criteria combined increased resistance to passive movement with at least one clinically relevant feature, such as pain, involuntary movements, or impaired active or passive function [21]. EUBoSS provides perhaps the clearest experimental example of a timely, target-triggered intervention strategy. Patients with severe upper-limb impairment were screened repeatedly during the first 6 weeks after stroke and received study treatment when abnormal stretch-related muscle activation was identified using surface electromyography during passive movement [17]. Thus, rather than administering BoNT-A at a predetermined post-stroke date, EUBoSS linked treatment initiation to the emergence of a detectable neural treatment target in a high-risk patient. Importantly, this approach remained embedded within a predefined early post-stroke window; it therefore illustrates how chronological and impairment-based criteria can be combined rather than treated as mutually exclusive approaches to timing.

Chronology nevertheless remains clinically relevant because the acute and early subacute phases overlap with a period of particularly dynamic spontaneous recovery and evolving biological processes after stroke [19]. However, the temporal profile of spontaneous biological recovery should not be equated with a time limit on neural plasticity. Experience- and practice-dependent neural reorganization and functional adaptation may continue throughout the late subacute and chronic phases and remain influenced by rehabilitation and environmental demands [19,20]. The stroke recovery timeline should therefore provide biological context for treatment decisions rather than function as an isolated criterion for BoNT-A initiation.

Translating the concept of timely treatment into clinical practice requires consideration of multiple interacting variables. Table 2 proposes a pragmatic clinical reasoning framework organized around the following domains: the focal neural treatment target; trajectory and risk of secondary complications; passive musculoskeletal status; symptoms and care burden; active function and motor recovery context; rehabilitation context; patient-centered goals; and treatment safety and feasibility. These variables are intended to support individualized decision-making rather than constitute a validated decision rule, and no single variable should automatically mandate injection.

Accordingly, the key clinical question is not simply “how early after stroke should BoNT-A be administered?” but rather “when does a focal neural treatment target become sufficiently clinically relevant, problematic, or prognostically important to justify focal chemodenervation within a goal-oriented rehabilitation plan?” Treatment should not be unnecessarily delayed until fixed contracture, chronic deformity, or substantial care burden has developed; conversely, every minimal or transient increase in resistance to passive movement should not automatically trigger injection. The most clinically meaningful paradigm may therefore be timely, target- and goal-guided BoNT-A treatment, in which chronology provides important biological context, but treatment initiation is determined by the emergence, clinical relevance, and modifiability of the individual treatment target.

## 7. Myth or Reality?

The answer depends less on how soon after stroke BoNT-A is administered than on how “early treatment” is defined and operationalized. If early treatment is interpreted as routine or indiscriminate injection during the first days or weeks after stroke based solely on chronology or an isolated increase in resistance to passive movement, then it remains unsupported by the available evidence. Such an approach could lead to unnecessary treatment of mild or transient abnormalities and would not account for the distinction between neural involuntary muscle overactivity, other manifestations of the upper motor neuron syndrome, and evolving non-neural musculoskeletal changes.

If, however, early treatment is understood as timely, target- and goal-guided BoNT-A treatment in appropriately selected patients, it is increasingly supported by the available evidence. This paradigm applies when focal involuntary muscle overactivity represents a clinically relevant and modifiable treatment target, or when, in selected high-risk patients, it is judged likely to contribute to clinically important secondary consequences. The strongest evidence concerns reduction in focal involuntary muscle overactivity and clinically measured resistance to passive movement, delay of symptomatic progression, slowing of passive range-of-motion loss and contracture development, and reduction in pain-related complications [9,15,17,18,21,22,23,24,25,26]. Randomized studies also provide reassurance that the early BoNT-A dosing regimens evaluated to date do not appear to interfere with spontaneous upper-limb motor recovery. However, a consistent additional effect on active motor recovery has not been demonstrated, and evidence for sustained functional independence, participation, and other long-term patient-centered outcomes remains less definitive. Observational associations suggesting advantages of earlier treatment should also not be interpreted as proof of a causal benefit of early versus delayed BoNT-A.

Accordingly, the debate should move beyond a rigid chronological threshold toward timely, target- and goal-guided treatment. Chronology remains relevant because the biological context of stroke recovery changes over time, but elapsed time from stroke should not determine treatment initiation in isolation. The more clinically meaningful approach is to identify patients at risk, determine whether a modifiable focal neural treatment target is present, assess its current or anticipated consequences, define individualized goals, and integrate BoNT-A within the rehabilitation plan when the expected clinical benefit justifies intervention. The variables proposed in Table 2 provide a pragmatic scaffold for this decision-making process while preserving individualized clinical judgment; they should not be interpreted as a validated treatment algorithm.

## 8. Conclusions

Early BoNT-A treatment for PSS is neither a myth nor an established universal rule. Current evidence supports a more nuanced interpretation: BoNT-A can be considered earlier in the post-stroke course when an appropriate focal neural treatment target is identified, but its clinical value depends on patient selection, treatment goals, the evolving contribution of neural and non-neural impairments, and integration within a multidisciplinary rehabilitation strategy.

Two complementary dimensions should guide treatment timing. First, the biological timeline of stroke recovery provides an important context. The first weeks to months after stroke, particularly the early subacute phase, overlap with a period of rapid spontaneous recovery and substantial biological change [19]. This may provide a relevant context in which to address clinically important focal involuntary muscle overactivity before fixed biomechanical limitations become established. However, the SRRR framework should not be interpreted as defining a BoNT-A-specific therapeutic window, and neural plasticity, rehabilitation-related change, and functional adaptation may continue into the late subacute and chronic phases [19,20]. Second, treatment timing should be linked to the emergence and clinical relevance of the treatment target rather than to chronology alone. BoNT-A should therefore be considered when focal involuntary muscle overactivity becomes clinically relevant, interferes with individualized goals, or is judged likely to contribute to important secondary consequences in an appropriately selected patient.

Future studies should move beyond reliance on resistance-based measures alone and should distinguish, where possible, neural involuntary muscle overactivity from non-neural contributions to passive resistance. Standardized definitions of “early” and “timely” treatment, as well as clinically relevant, symptomatic, and disabling spasticity, are needed to improve comparability across studies. Adequately powered prospective trials should determine whether different treatment triggers or timing strategies influence long-term active function, participation, quality of life, caregiver burden, goal attainment, and cost-effectiveness, while carefully documenting baseline motor impairment, recovery potential, BoNT-A treatment parameters, and rehabilitation type, timing, and dose.

Until such evidence is available, the most defensible interpretation is that early BoNT-A treatment represents an emerging clinical reality when applied as a timely, target- and goal-guided intervention in appropriately selected patients, rather than as a routine treatment triggered by time from stroke or by any isolated increase in resistance to passive movement.

## Figures and Tables

**Table 1:** Representative studies informing the timing of BoNT-A treatment after stroke.

Study/Design	Population or Treatment Trigger	Timing from Stroke	Comparator/Timing Definition	Timing-Relevant Findings
Cousins et al. [22]; phase II double-blind pilot RCT	Severe arm-function deficit; treatment given before clinically evident spasticity was considered to warrant routine treatment.	Within 3 weeks	Quarter-dose or half-dose BoNT-A vs. saline placebo	Arm function improved in all groups; no whole-group advantage of active treatment and no evidence of impaired recovery. A prespecified ARAT = 0 subgroup favored active treatment.
ONTIME [21]; phase IV double-blind pilot RCT	First stroke; MAS ≥ 2 in a primary targeted upper-limb muscle group. Patients could be asymptomatic or have ≥1 symptomatic feature in addition to increased resistance.	2–12 weeks; median 5.86 weeks	AbobotulinumtoxinA 500 U vs. placebo; re-injection required MAS ≥ 2 plus ≥1 symptomatic feature	BoNT-A prolonged time to re-injection/symptomatic progression criteria compared with placebo.
EUBoSS [18]; phase II double-blind RCT	First stroke; severe upper-limb impairment (ARAT grasp ≤ 2). Randomization occurred when repeated surface EMG during passive stretch first detected abnormal muscle activity.	Within 42 days; mean 18 days	OnabotulinumtoxinA vs. saline placebo; both groups received wrist-extensor electrical stimulation	Reduced EMG-defined spastic muscle activity, slowed contracture development, reduced splint use, and did not appear to hinder arm-function recovery.
early-BIRD [23]; prospective observational routine-practice study	Clinically relevant post-stroke upper-limb spasticity treated according to routine practice; cohort included BoNT-naïve and previously treated patients.	Early-start mean 3.74 months; medium 20.11; late 144.24 months	Quartiles of time from stroke to treatment in the study	All timing groups improved; reductions in MAS-based stiffness descriptively favored earlier treatment but between-group differences were not significant.
Picelli et al. [9]; multicenter longitudinal cohort	First-ever stroke < 12 months; BoNT-A-naïve; PSS with MAS ≥ 1+ and antagonist strength ≤ 2/5 MRC; fixed contracture excluded.	≤90 days vs. >90 days (all < 12 months)	Timing groups; no placebo comparator	Earlier treatment was associated with greater MAS-based stiffness reduction at 4 and 12 weeks.
Patel et al. [25]; retrospective pooled individual-data cohort	Adults with PSS who received ≥1 BoNT-A injection and had Goal Attainment Scaling at baseline and approximately 12 weeks.	Early < 1 year (median 0.5 year) vs. late ≥ 1 year (median 5.4 years)	Retrospective early-vs-late timing groups	The early cohort showed greater improvement in goal-attainment scores at 12 weeks.

Abbreviations: ARAT, Action Research Arm Test; BoNT-A, botulinum toxin type A; EMG, electromyography; MAS, Modified Ashworth Scale; MRC, Medical Research Council; PSS, post-stroke spasticity; RCT, randomized controlled trial. The table summarizes differences in study design, eligibility criteria, treatment triggers, post-stroke timing definitions, comparators, and timing-related outcomes. Observational timing comparisons should not be interpreted as randomized early-versus-delayed treatment comparisons.

**Table 2:** Proposed clinical variables to consider when deciding whether BoNT-A treatment is timely in post-stroke spasticity.

Clinical Domain	What to Assess	Why It Matters for Timing	Potential Implication for BoNT-A Planning
Target impairment	Focal involuntary muscle activity, affected pattern/muscles, dynamic resistance to passive movement, and whether a neural component is clinically plausible.	BoNT-A acts on focal muscle activation; resistance caused predominantly by fixed musculoskeletal change is less modifiable by chemodenervation.	Confirm that the intended target is focal and modifiable; consider neurophysiological assessment when the clinical picture is equivocal.
Trajectory and risk	Time of onset, recent progression, sustained abnormal posturing, recurrent involuntary movements, severity of paresis, and emerging loss of range.	Rapidly evolving clinically relevant PSS may increase the risk of secondary complications.	A progressive trajectory may favor prompt assessment rather than waiting for chronic deformity.
Passive musculoskeletal status	Passive range of motion, end-feel, passive stiffness, muscle-tendon extensibility, early shortening, fixed contracture, or bony deformity.	Potential to prevent or slow biomechanical complications is greatest before changes become fixed.	Define realistic passive goals; fixed deformity may reduce the expected benefit of BoNT-A alone.
Symptoms and care burden	Pain, difficulty with hygiene, dressing, positioning, limb care, skin protection, or caregiver assistance.	These features indicate that PSS is clinically relevant rather than an isolated examination finding.	Prioritize symptom- and care-related goals that are plausibly responsive to focal treatment.
Active function and motor recovery context	Residual strength, selective motor control, task performance, and whether the target muscle contributes useful voluntary activity.	Treatment should reduce unwanted activation without unnecessarily weakening functionally useful muscle activity.	Select muscles and dose according to the balance between problematic overactivity and preserved voluntary function.
Rehabilitation context	Current therapy goals and dose, opportunities for task practice, positioning/orthotic strategies, and caregiver-supported practice.	The functional value of reduced unwanted activity depends partly on how it is integrated with rehabilitation.	Coordinate injection timing with a goal-oriented rehabilitation plan.
Patient-centered goals	Priorities for active function, passive function, pain, mobility, participation, self-care, and caregiver burden.	Clinical relevance is defined by impact on the person, not by a stiffness score alone.	Proceed when goals are meaningful, specific, and reasonably attributable to the targeted muscle overactivity.
Safety and feasibility	Contraindications, dose and muscle-selection constraints, injection access, prior BoNT-A response where applicable, and ability to review outcomes.	Timing is only appropriate when the expected benefit-risk balance and follow-up are acceptable.	Individualize treatment plan and reassessment interval; defer or modify treatment when safety or feasibility is limiting.

This table is a pragmatic framework proposed by the authors on the basis of the evidence and goal-oriented principles discussed in. It is not a validated decision rule, and no single variable should be considered an automatic indication for injection.

## Data Availability

No new data were created or analyzed in this study. Data sharing is not applicable to this article.

## Abbreviations

The following abbreviations are used in this manuscript:

PSS	Post-stroke spasticity
BoNT-A	Botulinum toxin type A
MAS	Modified Ashworth scale
SRRR	Stroke Recovery and Rehabilitation Roundtable

## References

[1] Sunnerhagen K.S., Opheim A., Alt Murphy M. (2019). Onset, time course and prediction of spasticity after stroke or traumatic brain injury. Ann. Phys. Rehabil. Med..

[2] Portilla-Jiménez M., Yang Y., Roh J., Rymer W.Z., Li S. (2026). Post-stroke abnormal synergies are a resultant behavior of the interplay of weakness, spasticity, and environmental demands. Neurorehabil. Neural Repair.

[3] Xing Y., Liu J., Wu L., Zhang K., Yang S. (2026). A narrative review of motor control dual pathways: The corticospinal and reticulospinal tracts in synergy and differentiation. Front. Neuroanat..

[4] Zeng S., Li L., Wu L., Li R., Tang X., Sun X., Lin S., Liu Z., Tang J., Liu Q. (2025). Excitation-inhibition imbalance in the motor control network: A key factor in post-stroke spasticity. Front. Hum. Neurosci..

[5] Francisco G.E., Pandyan A., Li S., Esquenazi A., Deltombe T., Baude M., Picelli A., Baguley I., Carda S., Baricich A. (2026). Redefining Spasticity: The Spasticity X Working Group Consensus Statement. Am. J. Phys. Med. Rehabil..

[6] Li S., Francisco G.E. (2015). New insights into the pathophysiology of post-stroke spasticity. Front. Hum. Neurosci..

[7] Francisco G.E., Deltombe T., Carda S., Reebye R., Draulans N., Picelli A., Biering-Sørensen B., Bensmail D., Verduzco-Gutierrez M., O’Dell M. (2026). Is spasticity a syndrome? A historical perspective on spasticity definitions and descriptions. Am. J. Phys. Med. Rehabil..

[8] Gracies J.M. (2005). Pathophysiology of spastic paresis. I: Paresis and soft tissue changes. Muscle Nerve.

[9] Picelli A., Santamato A., Cosma M., Baricich A., Chisari C., Millevolte M., Del Prete C., Mazzù I., Girardi P., Smania N. (2021). Early botulinum toxin type A injection for post-stroke spasticity: A longitudinal cohort study. Toxins.

[10] Zeng H., Chen J., Guo Y., Tan S. (2021). Prevalence and risk factors for spasticity after stroke: A systematic review and meta-analysis. Front. Neurol..

[11] Lundström E., Terént A., Borg J. (2008). Prevalence of disabling spasticity 1 year after first-ever stroke. Eur. J. Neurol..

[12] Simpson D.M., Hallett M., Ashman E.J., Comella C.L., Green M.W., Gronseth G.S., Armstrong M.J., Gloss D., Potrebic S., Jankovic J. (2016). Practice guideline update summary: Botulinum neurotoxin for the treatment of blepharospasm, cervical dystonia, adult spasticity, and headache: Report of the Guideline Development Subcommittee of the American Academy of Neurology. Neurology.

[13] Wissel J., Ward A.B., Erztgaard P., Bensmail D., Hecht M.J., Lejeune T.M., Schnider P. (2009). European consensus table on the use of botulinum toxin type A in adult spasticity. J. Rehabil. Med..

[14] Ashford S., Turner-Stokes L., Allison R., Duke L., Moore P., Bavikatte G., Kirker S., Ward A.B., Bilton D. (2018). Spasticity in Adults: Management Using Botulinum Toxin. National Guidelines.

[15] Rosales R.L., Efendy F., Teleg E.S.A., Delos Santos M.M.D., Rosales M.C.E., Ostrea M., Tanglao M.J., Ng A.R. (2016). Botulinum toxin as early intervention for spasticity after stroke or non-progressive brain lesion: A meta-analysis. J. Neurol. Sci..

[16] Wissel J., Ri S., Kivi A. (2023). Early versus late injections of Botulinumtoxin type A in post-stroke spastic movement disorder: A literature review. Toxicon.

[17] Lindsay C., Ispoglou S., Helliwell B., Hicklin D., Sturman S., Pandyan A. (2021). Can the early use of botulinum toxin in post stroke spasticity reduce contracture development? A randomised controlled trial. Clin. Rehabil..

[18] Lindsay C., Philp F., Pandyan A.D. (2026). Early use of botulinum toxin in post-stroke spasticity has the potential to prevent post-stroke upper limb pain—A secondary analysis of the EUBoSS randomised controlled trial. Toxins.

[19] Bernhardt J., Hayward K.S., Kwakkel G., Ward N.S., Wolf S.L., Borschmann K., Krakauer J.W., Boyd L.A., Carmichael S.T., Corbett D. (2017). Agreed definitions and a shared vision for new standards in stroke recovery research: The Stroke Recovery and Rehabilitation Roundtable taskforce. Int. J. Stroke.

[20] Michielsen M.E., Selles R.W., van der Geest J.N., Eckhardt M., Yavuzer G., Stam H.J., Smits M., Ribbers G.M., Bussmann J.B.J. (2011). Motor recovery and cortical reorganization after mirror therapy in chronic stroke patients: A phase II randomized controlled trial. Neurorehabil. Neural Repair.

[21] Rosales R.L., Balcaitiene J., Berard H., Maisonobe P., Goh K.J., Kumthornthip W., Mazlan M., Latif L.A., Delos Santos M.M.D., Chotiyarnwong C. (2018). Early abobotulinumtoxinA (Dysport^®^) in post-stroke adult upper limb spasticity: ONTIME pilot study. Toxins.

[22] Cousins E., Ward A., Roffe C., Rimington L., Pandyan A. (2010). Does low-dose botulinum toxin help the recovery of arm function when given early after stroke? A phase II randomized controlled pilot study to estimate effect size. Clin. Rehabil..

[23] Wissel J., Fheodoroff K., Hoonhorst M., Müngersdorf M., Gallien P., Meier N., Hamacher J., Hefter H., Maisonobe P., Koch M. (2020). Effectiveness of abobotulinumtoxinA in post-stroke upper limb spasticity in relation to timing of treatment. Front. Neurol..

[24] Picelli A., Santamato A., Cosma M., Baricich A., Chisari C., Millevolte M., Del Prete C., Mazzù I., Di Censo R., Smania N. (2025). Early botulinum toxin type A injection may improve motor recovery in patients with post-stroke spasticity: A secondary analysis from a longitudinal cohort study. Toxins.

[25] Patel A., Zhang J., Page S., Harding S., Beneteau M., Navickas C., Esquenazi A. (2026). Impact of early versus late treatment with botulinum toxin A on goal attainment in post-stroke spasticity: A retrospective cohort study. Toxins.

[26] Fheodoroff K., Waeschle B., Schuetzer M., Comes G., Wissel J. (2026). Early recognition of post-stroke spasticity: The I-REFER study. Front. Neurol..

